# Long-term follow-up after cancer rehabilitation using high-intensity resistance training: persistent improvement of physical performance and quality of life

**DOI:** 10.1038/sj.bjc.6604433

**Published:** 2008-06-24

**Authors:** I C De Backer, G Vreugdenhil, M R Nijziel, A D Kester, E van Breda, G Schep

**Affiliations:** 1 Department of Sports Medicine, Máxima Medical Centre, Veldhoven, The Netherlands; 2 Department of Internal Medicine, Máxima Medical Centre, Veldhoven and Eindhoven, The Netherlands; 3 Department of Methodology and Statistics, Maastricht University, Maastricht, The Netherlands; 4 Department of Movement Science, Nutrition and Toxicology Research Institute Maastricht (NUTRIM), Maastricht University, Maastricht, The Netherlands

**Keywords:** exercise, fatigue, long-term effects, muscle strength, quality of life, rehabilitation

## Abstract

The short-term beneficial effects of physical rehabilitation programmes after cancer treatment have been described. However, little is known regarding the long-term effects. The purpose of this study was to investigate the long-term effects of high-intensity resistance training compared with traditional recovery. A total of 68 cancer survivors who completed an 18-week resistance training programme were followed for 1 year. During the 1-year follow-up, 19 patients dropped out (14 due to recurrence of cancer). The remaining 49 patients of the intervention group were compared with a group of 22 patients treated with chemotherapy in the same period but not participating in any rehabilitation programme. Outcome measures were muscle strength, cardiopulmonary function, fatigue, and health-related quality of life. One year after completion of the rehabilitation programme, the outcome measures in the intervention group were still at the same level as immediately after rehabilitation. Muscle strength at 1 year was significantly higher in patients who completed the resistance training programme than in the comparison group. High-intensity resistance training has persistent effects on muscle strength, cardiopulmonary function, quality of life, and fatigue. Rehabilitation programmes for patients treated with chemotherapy with a curative intention should include high-intensity resistance training in their programme.

Oncologists and scientists have made substantial progress in cancer treatment in the last few decades. Currently, the average 5-year survival rate is approaching 60% for female and 46% for male patients (Signaleringscommissie Kanker, 2004). Between 2000 and 2015 the number of cancer survivors in the Netherlands is expected to double. Not only improved medical treatment but also greying of the population and a longer life expectancy are contributing to increased cancer prevalence worldwide. As both the number of cancer survivors and the length of their survival are increasing, long-term health issues related to cancer and its treatment are becoming more important (Demark-Wahnefried *et al*, 2005; Ganz, 2005).

Cancer treatment is associated with substantial psychosocial and physical side effects, including muscular atrophy, weight changes, decreased resistance, depression, fatigue, and an overall decrease in quality of life (Berglund *et al*, 1991; Dimeo *et al*, 1997, 2004; Courneya and Friedenreich, 1999; Courneya, 2003; Wagner and Cella, 2004). Furthermore, cancer survivors are at increased risk for cancer recurrence and for secondary effects, such as cardiovascular disease, diabetes, obesity, osteoporosis, and functional decline (Demark-Wahnefried *et al*, 2005). In several cross-sectional and intervention studies in healthy populations and in patients with chronic diseases, regular physical activity is associated with enhanced health and reduced risk of all-cause mortality (Blair *et al*, 1995, 1996; Roberts and Barnard, 2005; Lindstrom *et al*, 2006).

Rehabilitation programmes are currently being incorporated more and more in the care of cancer patients as well (Lucia *et al*, 2003; Galvao and Newton, 2005; Knols *et al*, 2005). Systematic review evidence shows that exercise in cancer survivors improves quality of life, cardiorespiratory fitness, physical functioning, and fatigue (Stevinson *et al*, 2004; Galvao and Newton, 2005; Knols *et al*, 2005; McNeely *et al*, 2006). However, several intervention studies incorporated in these reviews have some shortcomings. First, most rehabilitation programmes are relatively short in duration (less than 12 weeks) (Irwin and Ainsworth, 2004). Second, in these programmes, patients were not stimulated to remain physically active *after* the programme. Finally, most studies use aerobic exercises such as walking or stationary cycling (Irwin and Ainsworth, 2004). Few studies incorporated resistance training in their programmes. A recent systematic review by Cheema *et al* (2007) located only 10 trials that used progressive resistance training in breast cancer patients. However, even this limited number of studies indicates that resistance training has a great potential to counteract side effects of cancer, such as muscle wasting, reduced bone mineral density, and fatigue (Cunningham *et al*, 1986; Ferrando *et al*, 1997; Twiss *et al*, 2001; Segal *et al*, 2003; Oldervoll *et al*, 2004; Ott *et al*, 2004; Galvao and Newton, 2005; Ohira *et al*, 2006).

As cancer rehabilitation is a relatively new area of research, published studies mainly report the short-term effects of exercise training. This is a major drawback, as physical and psychological impairment may persist for many years after cancer treatment (Berglund *et al*, 1991; Dimeo, 2001). Only four training studies in cancer patients reported data of long-term follow-up. Two of them involved a home-based training programme and one study used only questionnaires to follow up the patients (Berglund *et al*, 1994; Carlson *et al*, 2006; Demark-Wahnefried *et al*, 2006; Thorsen *et al*, 2007). The fourth study examined the effects of supervised training after allogeneic stem cell transplantation (Carlson *et al*, 2006).

Our study investigated the long-term effects of high-intensity resistance training on muscle strength, cardiopulmonary function, fatigue, and quality of life in a more general population of cancer patients after chemotherapy. To distinguish the observed long-term effects from spontaneous recovery, a comparison was performed with a similar group of patients who did not participate in a rehabilitation programme. We hypothesised that cancer patients benefit from high-intensity resistance training in terms of muscle strength, cardiopulmonary function, fatigue, and quality of life immediately after rehabilitation and 1 year after completing the rehabilitation programme.

## Methods

### Study design and patient selection

This study was conducted in two teaching hospitals, Máxima Medical Centre, Eindhoven (hospital 1) and Máxima Medical Centre, Veldhoven (hospital 2). The project was approved by the Ethical Review Committee of the Máxima Medical Centre, and informed consent was obtained from all patients. Eligibility criteria included histologically confirmed cancer with no indication of recurrent or progressive disease, age between 25 and 70 years, chemotherapy with curative intention administered between January 2001 and December 2003, and completion of surgical treatment or radiotherapy. Patients suffering from other serious diseases that might hamper physical performance capacity, for example, heart failure, COPD, and neurological disorders, were excluded.

**Figure 1:**
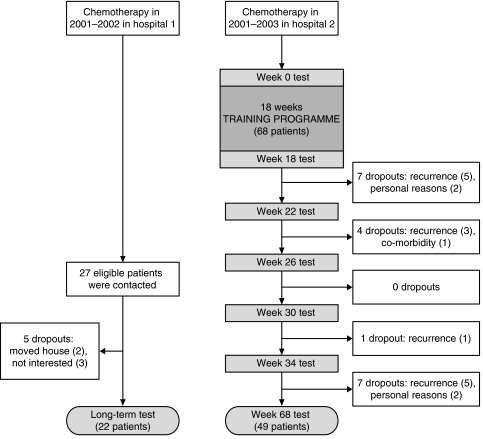
Flow chart of the study.

From 2001 onwards, rehabilitation using a high-intensity resistance training programme was implemented as standard medical care after chemotherapy in hospital 2. Medical oncologists recruited all eligible patients treated with chemotherapy with a curative intention. These patients were assigned to the intervention group and were prospectively followed from the start of the rehabilitation programme up to 12 months after completing the programme. The comparison group, treated in hospital 1, was a similar group of cancer patients who underwent chemotherapy in the same period as the intervention group. This group of patients was not offered any exercise or rehabilitation programme. The same inclusion and exclusion criteria were applied as in the intervention group. The oncologists and the patients in hospital 1 were not aware of the benefit of the rehabilitation programme in hospital 2 in 2001 and 2002. Figure 1 shows the flowchart of the study and the patient selection. One year after completion of the training programme, 49 consecutive patients were included in the follow-up. Twenty-two patients were included in the comparison group. These patients did not exercise under supervision. There was no further information from these patients about their activity level. The characteristics of these patients are shown in Table 1.

### Training intervention

The 18-week training programme consisted of high-intensity resistance and interval training. To counteract bias resulting from spontaneous recovery after chemotherapy, training started not earlier than 6 weeks after completing chemotherapy. The patients trained in groups of six to eight persons on specialised resistance training equipment and on bicycle ergometers under the supervision of physical therapists. During the first 12 weeks, patients were trained twice a week. In the last six weeks, patients were trained once a week.

#### High-intensity resistance training

The resistance programme consisted of six exercises targeting the large muscle groups as follows: (1) vertical row (focusing on longissimus, biceps brachii, rhomboideus); (2) leg press (quadriceps, glutei, gastrocnemius); (3) bench press (pectoralis major, triceps); (4) pull over (pectoralis, triceps brachii, deltoideus, trapezius); (5) abdominal crunch (rectus abdominis); and (6) lunge (quadriceps, glutei, hamstrings). First, resistance exercises were performed at 65–80% of one-repetition maximum (1-RM) and consisted of two sets of 10 repetitions. After the 12th week, the emphasis shifted from muscle strength to muscle endurance involving training with less resistance (35–40% of 1-RM) but more (20) repetitions. Every 4 weeks the training progress was evaluated, and the result was adjusted by means of a 1-RM test.

#### Interval training

Interval training consisted of cycling two times for 8 min, before and after the resistance exercises. In the first 8 weeks, those 8 min consisted of alternating 30 s at 65% of the maximal short exercise capacity (MSEC) and 60 s at 30%. A steep ramp test was performed to determine the MSEC. After 30 s of cycling at 25 W, the load was increased by 25 W every 10 s until exhaustion. From week 9, those 8 min consisted of alternating 30 s at 65% and 30 s at 30% of the MSEC. Results of the steep ramp test were described in a previous publication (De Backer *et al*, 2007a).

#### Follow-up

At the end of the rehabilitation programme (week 18), patients were advised by a sports physician to continue physical activity at home. These personalised advices were based on the patient's individual interests and motivation. In the follow-up period, there were five appointments (week 22, 26, 30, 34, and 68) with a physical therapist to encourage the patients to stay active and to perform a muscle strength test. In week 68, cardiopulmonary function, Multidimensional Fatigue Inventory (MFI), and health-related quality of life (HRQOL) were reported along with muscle strength.

### Outcome measures

#### Muscle strength

For the determination of muscle strength, the *indirect* 1-RM test was used (Sale and MacDougall, 1981; Mayhew *et al*, 1995). One-repetition maximum is the maximum amount of weight that can be lifted once. Indirect 1-RM values were calculated from the Brzycki's equation (Sale and MacDougall, 1981; Mayhew *et al*, 1995). One-repetition maximum is stated in kilograms in proportion to body weight. Muscle groups were tested with the resistance equipment that was also used for the training (leg press, vertical row, bench press, lunge, pull over, and abdominal crunch). This test was performed seven times: at the start (week 0) and the end of the programme (week 18) and in the follow-up period (weeks 22, 26, 30, 34, and 68).

#### Cardiopulmonary function

Cardiopulmonary function was assessed by cardiopulmonary exercise testing, which was performed on a cycle ergometer (Corival, Lode, The Netherlands). Expired gases were collected and analysed breath by breath for O_2_, CO_2_, and volume. Electrocardiogram was continuously monitored. Patients were instructed and encouraged to continue exercise until exhaustion. The test was ended if patients were unable to maintain the required pedalling frequency of 70 r.p.m. At the end of the test, peak oxygen consumption (peak *V*O_2_), peak power output, and peak heart rate were registered. Ventilatory threshold was determined by using the oxygen equivalent method (Wasserman *et al*, 1999). In addition, cardiopulmonary exercise testing was used to identify potential cardiopulmonary limitations caused by cardiotoxic (e.g. anthracyclins) or pulmotoxic (e.g. bleomycin) medications or by radiation therapy to the breast (Dimeo, 2001; Winningham, 2001; Myers, 2005).

This test was performed before (week 0) and after the training programme (week 18) and in week 68 according to the standard protocol (ERS Task Force on Standardization of Clinical Exercise Testing and European Respiratory Society, 1997).

#### Fatigue

The MFI is a questionnaire consisting of 20 statements for which the person has to indicate on a 7-point scale the extent to which the particular statement applies to him or her. The statement refers to aspects of fatigue experienced during the previous few days. Higher scores indicate a higher degree of fatigue. This self-report instrument consists of five subscales based on different dimensions: general fatigue, physical fatigue, reduced activity, reduced motivation, and mental fatigue. This questionnaire was completed in weeks 0, 18, and 68.

#### Health-related quality of life

Quality of life was assessed using the European Organisation for Research and Treatment of Cancer Core Quality of Life Questionnaire C30 (EORTC QLQ-C30). This questionnaire has a high reliability and validity (Aaronson *et al*, 1993; Groenvold *et al*, 1997). The EORTC QLQ-C30 encompasses 30 items divided into six functional scales (physical, role, cognitive, emotional and social functioning, and global quality of life), three symptom scales (fatigue, nausea, and pain), and six individual items. This questionnaire was completed in weeks 0, 18, and 68.

### Statistical analyses

In the training group, dropouts and patients who continued the study were analysed for differences in gender, age, cancer diagnosis, time from last treatment, and initial muscle strength by means of *χ*^2^ tests or independent samples *t*-tests.

*χ*^2^ Tests for categorical data and independent samples *t*-tests for continuous data were used to examine group differences in terms of gender, age, cancer diagnosis, time between completion of treatment, and long-term outcome between the training group and the comparison group.

A repeated measure analysis (SPSS mixed linear) was used to assess differences in muscle strength between seven different time points (weeks 0, 18, 22, 26, 30, 34, and 68). *Post hoc* Bonferroni correction was used as a protection against Type I error.

Paired sample *t*-tests were used to test the significance of changes in mean scores for cardiopulmonary function, fatigue, and HRQOL from baseline (week 0) to post-intervention (week 18), post-intervention to week 68, and week 0 to week 68. The last measured values for the dropouts were used for the week 68 test (intention-to-treat analysis).

Independent sample *t*-tests were used to analyse differences in muscle strength, cardiopulmonary function, HRQOL, and fatigue between the training group and the comparison group.

All statistical analyses were performed using the statistics program SPSS (version 13.0).

## Results

### Adherence and baseline characteristics

In the intervention group, 68 patients were monitored for 12 months after the rehabilitation programme. Fourteen patients were excluded because of cancer recurrence, and one patient was excluded because of serious co-morbidity. Four patients left during the follow-up period for personal reasons and were considered non-adherent dropouts, resulting in a dropout rate of 6%. Of the 27 patients who proved eligible to participate in the comparison group, 22 took part in the exercise tests and completed the questionnaires, resulting in a dropout rate of 23%. There were no significant differences in gender, age, and cancer diagnosis between the intervention and the comparison groups. Moreover, there were no significant differences in gender, age, and cancer diagnosis between the dropouts and those patients completing the study. The time interval between last treatment and long-term test was shorter in the training group than in the comparison group (96 *vs* 169 weeks, *P*<0.01).

### Long-term effects on muscle strength and differences in muscle strength between the training and the comparison groups

**Figure 2:**
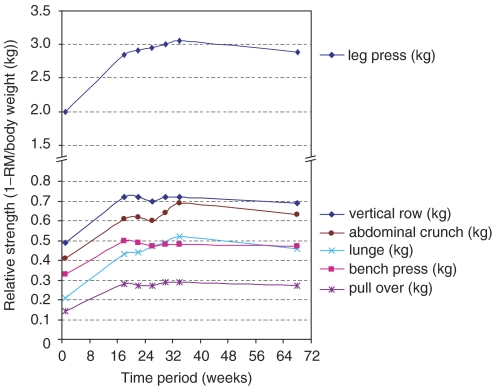
Muscle strength from start of rehabilitation up to 12 months after completing rehabilitation.

Table 2 shows the test results at baseline, post-rehabilitation, and after long-term follow-up (week 68) in the intervention group and the comparison group. Muscle strength improved significantly after training. Repeated measure analysis shows that the improvement of muscle strength was maintained in the long term. There were no significant differences between long-term measurements and post-rehabilitation in all resistance exercises. Figure 2 shows the progress in muscle strength in all exercises at seven different time points. After 18 weeks of training, muscle strength stabilises until week 68. All 1-RM test results were significantly higher in the intervention group than in the comparison group for vertical row (50%), leg press (33%), bench press (57%), pull over (100%), lunge (119%), and abdominal crunch (37%).

### Long-term effects on cardiopulmonary function and differences between the training and the comparison groups

Table 2 shows the data of cardiopulmonary outcomes. There was a significant effect of training on the peak oxygen consumption (+12%), maximal workload (+15%), peak heart rate (+3%), and ventilatory threshold (+16%). This effect was maintained in the long term, as shown by the fact that there were no significant differences in cardiopulmonary function between week 18 and week 68. Results of the comparison group were not significantly different from those of the intervention group.

### Long-term effects on fatigue and quality of life and differences between the training and the comparison groups

Table 2 shows the data of different subscales of fatigue (MFI) and quality of life (HRQOL). All subscales of MFI, except for reduction in motivation, improved significantly after training. The improvement in the fatigue outcome measures persisted 1 year after completing the training programme. However, in the long term, there were no differences between the comparison group and the intervention group in MFI. Health-related quality of life also improved significantly post-treatment, and this effect continued in the long term. There were no differences between the training group and the comparison group on all subscales of HRQOL.

## Discussion

This is the first study that describes the long-term effects of a high-intensity resistance training programme in cancer patients. The tolerance and effects noted immediately post-rehabilitation have already been published (De Backer *et al*, 2007b). After completion of the programme, repeated testing showed a continuation and stabilisation of the muscle strength level (Figure 2) and peak *V*O_2_. Muscle strength was significantly higher in patients who completed the resistance training programme than in the comparison group. Questionnaire outcomes indicate an overall improvement immediately after rehabilitation on several scales of quality of life and of fatigue, especially physical fatigue. This improvement was also maintained 1 year later. However, there were no differences in quality of life, fatigue, and peak *V*O_2_ between the intervention and the comparison groups.

There is preliminary evidence that physical activity plays a role in the primary and secondary prevention of cancer (Stein and Colditz, 2004; Holmes *et al*, 2005). Several randomised, controlled trials have examined the short-time effects of physical activity after the diagnosis of cancer. Overall, most studies demonstrated that physical training programmes had beneficial effects on cancer patients' physical or psychosocial capacity and, as a consequence, on their quality of life (Lucia *et al*, 2003; Knols *et al*, 2005). Despite the mounting evidence of the significance of physical activity after cancer treatment, there are presently only four studies that have assessed the long-term effects of physical rehabilitation programmes.

Two studies examined the long-term effect of a home-based training programme (Demark-Wahnefried *et al*, 2006; Thorsen *et al*, 2007). Demark-Wahnefried *et al* evaluated a 6-month home-based diet and exercise programme (telephone counselling and mailed materials) by telephone interview at 6 and 12 months after the start. This study included only older breast and prostate cancer patients (⩾65 years). In the long term (12 months), there were no significant differences between the intervention and the comparison groups in physical functioning, diet quality index, fatigue, and energy expenditure as measured by telephone questionnaires (Demark-Wahnefried *et al*, 2006). Thorsen *et al* recently published their long-term data of a randomised study of a 3-month home-based training programme. In contrast to our study, the favourable effect on cardiopulmonary functioning could not be sustained during a 12-month follow-up. The researchers concluded that for a longer-lasting effect over time, a longer intervention period and more intense exercise were needed (Thorsen *et al*, 2007). Another possible explanation for the lack of long-term effects is the supervised training used in our study rather than the home-based training programmes used in the previous studies. The multiple visits after the programme for testing may have contributed to the persistent effect in our programme.

The third study that examined the long-term effects of training in cancer patients is by Carlson *et al*. This study examined the effects of supervised aerobic exercise training after 12 months in 12 patients after allogeneic haematopoietic stem cell transplantation. Significant improvements in fatigue, ventilatory threshold, and stroke volume were found (Carlson *et al*, 2006). The fourth study of Berglund *et al* evaluated a training programme over a 1-year follow-up period. The intervention group improved significantly more than the comparison group in physical strength and physical activity levels, both of which were evaluated by means of questionnaires. In our study, the cardiopulmonary function and muscle strength were assessed by exercise tests (peak *V*O_2_ and 1-RM), which are more valid and reliable outcome measures.

As cancer rehabilitation is a relatively new area of research, there is no consensus about the optimal type of training in a rehabilitation programme. Exercise interventions vary considerably from brief instructions of home-based exercises to highly structured, supervised exercise sessions on strength equipment (Conn *et al*, 2006). It is remarkable that most studies only used aerobic exercises such as walking or stationary cycling in their rehabilitation programmes (Courneya *et al*, 2003), as one of the side effects of cancer and its treatment is muscle atrophy (Argiles *et al*, 2005). To improve muscle strength and increase muscle mass, resistance training is more important than aerobic exercise. Also, considerable evidence now suggests that the ability to perform physical tasks in daily life is determined by a threshold level of muscular resistance (Brill *et al*, 2000). As a consequence, resistance training seems to be the programme of choice for regaining muscle strength and, in this way, improving activities of daily living and HRQOL (Brill *et al*, 2000). In addition, in cancer patients, muscle strength is related to quality-of-life aspects with correlations ranging from 0.47 to 0.75 (De Backer *et al*, 2007b).

The results of the questionnaires, HRQOL, and MFI, did not differ between the two groups. A possible explanation for this is the high outcome values in the questionnaires. It is most likely that a ceiling effect is reached a rather long time after cancer treatment. Cancer patients may be satisfied with their ‘survival status’ and score high in all QOL questionnaires despite existing limitations and complaints. This experience is familiar to other researchers (van de Poll-Franse *et al*, 2006). Also, the significant difference in time span since the last treatment between the two groups, in favouring the comparison group, could be a reasonable explanation. Although in our study the mean level of quality of life is high, the large ranges for quality-of-life outcomes suggest that a subgroup of survivors may report lower quality-of-life levels.

Our study design has some shortcomings. First, the different time intervals between last treatment and long-term test for the comparison group (169 weeks) and the training group (96 weeks). However, we expect that this longer time span (if it has caused bias) will be in favour of the comparison group because this group has more time for spontaneous recovery after intensive treatment. Second, the lack of baseline measurements in the comparison group is a limitation of the current study. These shortcomings could be overcome by a prospective, randomised, controlled clinical trial. However, such a study design is not practical for exercise intervention trials. First, patients cannot be blinded to their treatment; only the outcome assessor can be blinded to group allocation. Second, when patients are participating in a training study and are randomised to the control or waiting group, they start to get more active spontaneously. Particularly in a group of cancer patients, subjects might come into contact with other subjects. Finally, the short-term benefits of exercise are recognised in research and clinics. It could be considered unethical to restrain patients from an exercise-containing rehabilitation programme. Therefore, we believe that our study design is appropriate in cancer rehabilitation research, especially when long-term effects are studied.

## Conclusion

This is the first study that describes the long-term effects of a high-intensity resistance training programme in cancer patients. Results indicate that after a 12-month follow-up, the beneficial effects on muscle strength, cardiopulmonary function, HRQOL, and fatigue were sustained. Muscle strength was significantly higher in the intervention group than in the comparison group. Based on these results, we suggest that guidelines for rehabilitation in oncology patients should include high-intensity resistance training.

## Figures and Tables

**Table 1:** Patient characteristics of the intervention and comparison groups

	**Intervention group (*n*=49)**		**Comparison group (*n*=22)**	
	***n***	**%**	***n***	**%**
*Gender*				
Male	9	18	4	18
Female	40	82	18	82
				
*Type of tumour*				
Breast	32	65	14	64
Ovarian	3	6	0	0
HL	4	8	2	9
NHL	3	6	3	14
Colorectal	5	10	3	14
Testis	2	4	0	0
				
*Treatment*				
Chemotherapy	49	100	22	100
+Radiotherapy	3	6	0	0
+Surgery	14	29	4	18
+Radiotherapy+surgery	28	57	17	77
				
*Chemotherapy*				
AC, breast	14	29	9	41
CMF, breast	7	14	3	14
FEC, breast	11	22	2	9
Carboplatin–paclitaxel, ovarian	3	6	0	0
ABVD/EBVP/BEACOPP, HL	4	8	2	9
CHOP/CVP, NHL	3	6	3	14
5-FU leucovorin, colorectal	5	10	3	14
BEP, testis	2	4	0	0
				
	**Mean**	**s.d.**	**Mean**	**s.d.**
*Age (years)*				
Mean	48	8	51	11
				
*Anthropometry*				
Height (cm)	170	7	173	8
Weight (kg)	78	11	77	14
				
*Time since last chemotherapy*				
Weeks between last chemotherapy and test week 68	96	26	169	26

ABVD=doxorubicin, bleomycin, vinblastine, dacarbazine; AC=adriamycin, cyclophosphamide; BEACOPP=bleomycin, etoposide, doxorubicin, cyclophosphamide, vincristine, procarbazine, prednisone; BEP=bleomycin, etoposide, cisplatin; CHOP=cyclophosphamide, doxorubicin, vincristine, prednisone; CMF=cyclophosphamide, methotrexate, fluorouracil; CVP=cyclophosphamide, vincristine, prednisone; EBVP=epirubicin, bleomycin, vincristine, prednisone; FEC=fluorouracil, epirubicin, cyclophosphamide; HL=Hodgkin's lymphoma; NHL=non-Hodgkin's lymphoma.

**Table 2:** Effects of training on muscle strength, cardiopulmonary function, fatigue, and quality of life on different time points

	**Baseline (week 0)**	**Post-rehabilitation (week 18)**	**Long-term (week 68)**	
**Outcome measure**	**Intervention group**	**Intervention group**	**Intervention group**	**Comparison group**
*Muscle strength (1-RM/kg)*				
Vertical row	0.48 (0.16)	0.69 (0.20)^a^	0.68 (0.20)^c^	0.46 (0.11)^e^
Leg press	1.96 (0.51)	2.79 (0.62)^a^	2.89 (0.74)^c^	2.20 (0.51)^e^
Bench press	0.30 (0.11)	0.46 (0.15)^a^	0.45 (0.15)^c^	0.30 (0.11)^e^
Pull over	0.12 (0.05)	0.25 (0.08)^a^	0.26 (0.10)^c^	0.13 (0.06)^e^
Lunge	0.20 (0.09)	0.42 (0.17)^a^	0.46 (0.16)^c^	0.21 (0.10)^e^
Abdominal crunch	0.39 (0.14)	0.60 (0.17)^a^	0.64 (0.18)^c^	0.46 (0.13)^e^
				
*Cardiopulmonary outcomes*				
Peak oxygen consumption (ml min^−1^ kg^−1^)	25.7 (6.3)	28.9 (6.7)^b^	29.3 (8.4)^d^	27.8 (5.6)
Peak power output (W kg^−1^)	2.0 (0.6)	2.3 (0.7)^b^	2.5 (0.8)^d^	2.2 (0.6)
Peak heart rate (beats per min)	167 (17)	172 (14)^b^	168 (20)^d^	165 (18)
Ventilatory threshold (ml min^−1^ kg^−1^)	19.2 (4.7)	22.3 (5.7)^b^	23.5 (7.1)^d^	22.2 (4.9)
				
*MFI*				
General fatigue	13.1 (4.5)	9.2 (4.1)^b^	9.9 (4.4)^d^	10.6 (4.4)
Reduced activity	11.8 (4.4)	8.1 (3.4)^b^	8.2 (3.8)^d^	8.8 (4.3)
Mental fatigue	10.0 (4.4)	8.8 (3.8)^b^	8.4 (4.0)^d^	7.9 (4.2)
Physical fatigue	13.7 (4.1)	8.1 (3.5)_b_	9.2 (4.0)^d^	9.9 (4.8)
Reduced motivation	8.7 (3.1)	7.7 (2.8)	7.8 (3.5)	8.4 (4.1)
				
*HRQOL*				
Physical functioning	72.4 (19.2)	84.2 (19.0)^b^	85.5 (18.5)^d^	81.8 (18.4)
Role functioning	60.3 (24.0)	79.5 (21.1)^b^	79.4 (22.5)^d^	83.3 (20.6)
Emotional functioning	75.6 (20.2)	85.7 (18.8)^b^	84.1 (18.4)^d^	81.3 (20.7)
Cognitive functioning	76.5 (24.4)	83.8 (21.5)	85.6 (17.3)^d^	82.6 (19.3)
Social functioning	68.4 (29.3)	82.5 (21.6)^b^	82.3 (24.2)^d^	84.1 (20.8)
Fatigue	43.6 (23.4)	22.0 (18.8)^b^	24.8 (22.0)^d^	29.8 (21.3)

HRQOL=health-related quality of life; MFI=multidimensional fatigue index; 1-RM=one-repetition maximum.

All data are means (s.d.).

a Significant difference, *P*<0.01, baseline and post-rehabilitation (repeated measure analyses).

b Significant difference, *P*<0.01, baseline and post-rehabilitation (paired *t*-tests).

c Significant difference, *P*<0.01, baseline and week 68 (repeated measure analyses).

d Significant difference, *P*<0.01, baseline and week 68 (paired *t*-tests).

e Significant difference, *P*<0.01, the intervention and comparison groups (independent sample *t*-tests).
